# Finite-Time Thermodynamics: Problems, Approaches, and Results

**DOI:** 10.3390/e27060649

**Published:** 2025-06-17

**Authors:** Anatoly M. Tsirlin, Alexander I. Balunov, Ivan A. Sukin

**Affiliations:** 1 System Analysis Research Center, Ailamazyan Program Systems Institute of RAS, 152021 Pereslavl-Zalessky, Russia; ivsukin@gmail.com; 2 Department of Cybernetics, Yaroslavl State Technical University, 150023 Yaroslavl, Russia

**Keywords:** irreversible thermodynamics, optimization, problem statements, thermodynamic balances, Carathéodory theorem, averaging, entropy production

## Abstract

In this manuscript, the typical problems of “finite-time thermodynamics”, their general methodology, and the general features of their solutions are considered. We also consider the role of minimal dissipation processes, the properties of the irreversibility index, and the consequences of its existence. A generalization of the Carathéodory theorem for averaged optimization problems corresponding to cyclic processes and the properties of optimal solutions following from it are given. The existence of the irreversibility index for economic macrosystems and their analogies to and differences from thermodynamic systems are proven.

## 1. Introduction. History of the Emergence of “Finite-Time Thermodynamics” and the Validity of Its Name

The development of thermodynamics, starting with the work of S. Carnot, is closely connected with extremal problems on the ultimate possibilities of thermodynamic systems. Carnot would hardly have obtained a solution to the problem of the ultimate efficiency of a heat engine if he had posed it in a mathematically rigorous manner, such as the following: *find a law T(t) of change in the temperature of the working fluid of a heat engine receiving heat from a source with temperature $T_{+}$ and giving the heat to a source with temperature $T_{-}$ such that the ratio ρ of the work produced to the heat taken from the hot source is maximal*. After all, one of the variables sought for in this problem is the duration of the cycle τ. The set of admissible values of this variable is limited only by the condition of non-negativity and therefore is not closed. Consequently, according to the Weierstrass theorem, which Carnot could not have known, the problem may not have a solution. Indeed, the maximum of ρ does not exist. The upper limit of the efficiency supremum is achieved in the limit as τ tends to infinity.

This feature was discovered to be characteristic of other extreme problems in thermodynamics (on the minimum work for separation of mixtures, on the efficiency of cycles for systems with sources of finite capacity, etc.). The solutions to these problems are reversible processes in which the exchange flows are arbitrarily close to zero; therefore, an arbitrarily long time is required to exchange a finite amount of substance or energy.

Note that a reversible Carnot heat engine can also have finite power but only in cases where the heat exchange coefficients between the heat sources and the working fluid of the heat engine (the dimensions of the engine) are arbitrarily large. In this case, the Carnot efficiency is the ratio of the “reversible power” to the heat flow taken from the hot source.

Apparently, the first problem in optimization thermodynamics was the “maximum power problem”, about a form of the heat engine cycle receiving heat from a source of infinite capacity with temperature $T_{+}$ and giving off heat to a source with temperature $T_{-}$, for which the power of the heat engine would be maximum. This was considered and solved soon after Carnot’s work; however, this result was not noticed. Much later, the problem of the maximum power cycle was solved in [1,2]. At that time, nuclear power began to develop. The cost of building nuclear power plants is very high, and the cost of fuel is much less than for thermal power plants. Under these conditions, obtaining maximum power is much more important than achieving maximum efficiency.

In the problem of maximum power, instead of the quantities of heat and work, heat and work flows appear within the kinetics of heat transfer when the dimensions of the heat engine are arbitrarily large. The authors of the above-mentioned and numerous other studies often solved this problem independently of each other but according to the same scheme:

1. Heat exchange flows in contact with each of the sources were assumed to be proportional to the difference in temperature between the source and the working fluid (Newtonian kinetics).
2. The desired cycle was assumed a priori to consist of two isotherms and two adiabats, similar to the Carnot cycle, and the temperatures of the working fluid in contact with the sources were selected based on the condition of maximum power, taking into account the law of conservation of energy and the fact that the entropy flow coming from a hot source must be equal to the entropy flow given to a cold source. The latter meant that the processes inside the working fluid were assumed to be reversible.

It was revealed that the maximum power of a heat engine in contact with reservoirs having temperatures $T_{+}$ and $T_{-}$ is equal to

(1) $$N_{max}=\frac{\alpha_{1}\alpha_{2}}{\alpha_{1}+\alpha_{2}}{\left(\sqrt{T_{+}}-\sqrt{T_{-}}\right)}^{2}.$$

where $\alpha_{1}$ and $\alpha_{2}$ are heat exchange coefficients in contact with sources. The efficiency of the maximum power cycle does not depend on the heat exchange coefficients and is equal to

(2) $$\eta =1-\sqrt{\frac{T_{-}}{T_{+}}}.$$

These works did not answer the following questions:

1. For what heat transfer kinetics does the maximum power cycle consist of two isotherms and two adiabats?
2. Is the efficiency of the maximum power cycle always independent of the heat transfer coefficients?
3. What is the shape of the heat engine cycle that has the maximum efficiency at a fixed power?

Answers to these questions were given in the works [3,4], in which the methods of averaged optimization developed in [5] were used. It was found that the following statements are true:

1. For any heat exchange kinetics satisfying the natural condition that the direction of heat flow coincides with the sign of the temperature difference of the contacting bodies, the maximum power cycle consists of two isotherms and two adiabats.
2. The efficiency corresponding to this cycle for kinetics that differ from Newtonian depends on the kinetic coefficients.
3. The cycle of a heat engine with maximum efficiency at a fixed power for arbitrary kinetics can consist of three isotherms and three adiabats. There, a condition was obtained under which the number of isotherms is equal to two.

The development of optimization thermodynamics from the ≪maximum power problem≫ to its current state began after the publication of the work of Curzon and Ahlborn [2]. This happened largely because the talented researchers P. Salamon, B. Andresen and K. H. Hoffmann ended up at the Department of Chemistry at the University of Chicago, headed by R.S. Berry. They realized that in real thermodynamic processes, thermal, mass transfer, chemical, etc., the factor of limited duration plays a crucial role. It is important to evaluate the capabilities of systems with an additional restriction on the duration of processes, i.e., in the class of irreversible processes. Thus, a new field of thermodynamics arose, which they called ≪Finite-time thermodynamics≫ (see [6,7,8,9] and many other publications).

The name ≪Finite-Time Thermodynamics≫ (FTT) has taken hold and is hard to abandon. However, this name cannot be considered successful. The overwhelming majority of problems on optimal irreversible processes do not contain time at all. The processes occur in stationary or cyclically operating systems. In the latter case, the indicators are averaged over the cycle time and time is not included in the problem after averaging.

Three classes of problems are typical:

1. Optimal periodic processes of limited duration
2. Optimal cyclic processes
3. Optimal processes in open stationary systems.

At the same time, for any class of problems, including problems on periodic processes, their conditions lead to non-zero flows due to their limited duration.

The correct name for this area of thermodynamics would be ≪Thermodynamics with non-zero flows≫. It emphasizes the features of all problems arising in it.

In order to be able to use the apparatus of equilibrium thermodynamics when solving problems that arise, the system is divided into several equilibrium subsystems, and irreversibility is determined only by the processes that occur when equilibrium subsystems come into contact with each other.

Here, thermodynamic systems should be understood as all systems consisting of a large number of interacting elements, each of which cannot be controlled. Control of such systems is possible only at the macro level and this control imposes the same conditions on all microelements. Such systems are called *macrosystems*. Examples of macrosystems may be economic systems, where microelements are sellers and buyers, individual ≪households.≫; social systems, where microelements are taxpayers, transport passengers, and others. An important class of macrosystems are *segregated* systems [10], whose elements (households, units in fluidized bed apparatuses, biomass in biochemical reactors, etc.) evolve under the influence of the environment. The parameters of the environment are determined by external influences and the average effect of the units.

All macrosystems are characterized by the division of the variables characterizing them into intensive and extensive. That implies the existence of processes that proceed spontaneously in one direction, without the expenditure of external resources, and in the other requiring the involvement of external resources for their implementation, i.e., the processes in such systems are irreversible. The irreversibility index for any processes in closed macrosystems increases. However, the form and the fact of the existence of such an index require proof for each type of macrosystem. For thermodynamic systems, such an indicator is entropy, for economic systems, the ≪utility function≫, the proof of the existence of which is given below. The irreversibility indicator is always the product of the flow and the driving force causing this flow. The sign of the flow coincides with the sign of the driving force, so their product is non-negative.

In macrosystems, there is a special subsystem, an *intermediary*. The intermediary (a reseller in economics, a working fluid in thermodynamics) reduces the growth rate of the irreversibility indicator and, due to this, extracts the target resource from the system (capital in economics, work in thermodynamics). For the intermediary to operate, a difference in the values of intensive variables in the subsystems is necessary. In economics, a difference in prices, in thermodynamics, a difference in temperatures or chemical potentials.

In the following sections, we will present the formulations of typical FTT problems, then the general methodology, and the results of their solution. Let us consider the mathematical features of solving problems of resource exchange and optimization of cyclic processes.

The present work is a review of some results obtained by the authors in solving FTT problems and in developing mathematical methods necessary for such a solution. Our goal is to show by examples that FTT has developed as a complete section of thermodynamics with its own methodology and class of problems, that it has allowed formulating and solving problems that could not even be posed within the framework of classical thermodynamics, that the FTT methodology is also applicable to other types of macrosystems, such as microeconomics, that FTT required the development of averaged optimization methods, and that there are unsolved problems in FTT.

Let us list these results:

1. Processes of minimum dissipation.
2. Construction of attainability sets of thermodynamic systems.
3. Extraction of maximum work from a non-uniform system in a limited time.
4. Limit capabilities of heat exchange systems.
5. Microeconomic analogies.
6. Optimality conditions for averaged problems and Lyapunov-type equations.

## 2. Statements and Solutions of Problems of FTT

### 2.1. The Problem of Maximum Possible Productivity

Each non-isolated thermodynamic system exchanges energy and material flows with its environment. In steady-state conditions, these flows can be stationary or periodic. In the latter case, the flow intensity is understood as its average intensity over a period. The mechanism of system operation (kinetics of heat and mass transfer, chemical reactions, etc.) establishes a connection between the incoming and outgoing flows. From the outgoing flows, a target flow can be identified or formed. Its intensity is the system’s productivity. The incoming flows form a cost flow. For a heat engine, the target flow is power. The cost flow is heat taken from a hot source.

The most studied types of macrosystems are thermodynamic. In the first section, we will present the formulations, methodology, and results of solving typical problems for such systems. In the second section we will focus on systems of an economic nature, and in the third we will prove the conditions that determine the type of optimal solution to problems with averaging, characteristic of macrosystems.

The problem of the maximum possible productivity of a thermodynamic system of arbitrary nature under certain constraints and the value of the cost flow that ensures this maximum productivity is a direct generalization of the maximum power problem.

This raises the following questions:

1. For which systems is productivity limited from above, and for which systems, by increasing the cost flow, can productivity be made arbitrarily large, and therefore the problem of maximum productivity has no solution?
2. There is a system of two or more thermodynamic reservoirs and a working fluid that contacts each of them in a steady state or alternately and produces a target flow. How should the contacts of the working fluid be organized to obtain the maximum value of the target flow? What should be considered the efficiency of such a system?
3. What will change if in problem 2, instead of reservoirs, there are sources of finite capacity? In particular, what is the maximum work that can be extracted in a closed thermodynamic system with a fixed duration? This problem coincides with the problem of calculating the exergy of a system in the particular case where the duration of the process is unlimited.
4. How to organize thermodynamic processes so that for a given average flow intensity increase in entropy of the system is minimal (minimum dissipation processes)?
5. In particular, what criterion should be used to evaluate the heat exchange process? How to organize the heat exchange process of two vector flows so that for a given heat load and total heat exchange coefficient the entropy production is minimal?
6. How to construct the region of realizable modes of a thermodynamic system with limited kinetic coefficients in a space along the axes of which the flow intensities are plotted.

The above formulations, of course, do not exhaust the problems in FTT, but they allow us to judge the nature and applied focus of the problems that arise. Note that in most of them the duration of the process does not figure.

### 2.2. General Methodology for Solving Problems of FTT, Dissipation

Thermodynamic systems are characterized by two types of variables: intensive and extensive. The first type do not change when combining subsystems if before combining they were equal in each subsystem (temperature, pressure, concentration …), while the second type are are added when combining such subsystems (volume, number of moles, internal energy …).

In this case, subsystems can be either *passive*, in which case intensive variables are determined by extensive variables and the equation of state, or *active* (the working fluid of a heat engine, or an absorption-desorption cycle), in which case intensive variables (they are controls) are selected to achieve one or another goal.

Let us first characterize the general scheme by which these and similar problems can be solved.

- The first step in studying the ultimate possibilities of thermodynamic systems is to compile balance relations for matter, energy, and entropy. The last of these relations will include a term characterizing the irreversibility of processes: entropy production rate σ. It is equal to the rate of growth of the entropy of the system. This term is equal to zero if all processes in the system are reversible, and greater than zero for irreversible processes. The non-negativity of dissipation, due to the equations of thermodynamic balances, determines a certain set of realizability in the parameter space of input and output flows.
- If additional conditions of finite duration of processes, given average flow intensity, and limited kinetic coefficients are imposed on the system, then the dissipation value that is minimally possible under these restrictions is found. In any real system $\sigma \geq \sigma_{min}$, which narrows the set of realizability. Now this set takes into account the kinetics of the processes as well as the dimensions of the installation through the heat and mass transfer coefficients.
- The third step is to obtain from the balance equations the relationship between one or another indicator of the system’s efficiency and the dissipation σ. As a rule, natural efficiency indicators monotonically deteriorate with the growth of σ and reach their limit values in a reversible process, which leads to estimates similar to the Carnot efficiency for processes of very different nature.
- Since in a complex system dissipation additively depends on dissipation in each of the elementary processes, an important stage of the study is to identify the conditions of minimal dissipation. Optimal organization of processes in a complex system comes down to coordinating individual processes of minimal dissipation with each other.

### 2.3. Thermodynamic Balances

Thermodynamic balances are a system of equations of material, energy, and entropy balances [11,12]. For simplicity, we will consider them for an open system. Some flows enter the system from outside, others are generated in the system. One of such flows generated in the system is entropy production, which is non-negative. This circumstance turns the entropy balance equation into an inequality. Together with the other equations, it identifies a region of feasibility in the space of flows, the boundary of which corresponds to reversible processes. If, however, in one way or another it was possible to solve the problem of the minimum possible entropy production $\sigma_{min}$ under the restrictions imposed on the process, then the equations of thermodynamic balances with the condition $\sigma \geq \sigma_{min}$ identify a region whose boundary is determined by processes of minimal dissipation. This region lies inside the region bounded by reversible processes.

**Open system.** Thermodynamic balances establish a connection between the flows of each substance, energy, and entropy that the system exchanges with the environment, as well as the occurrence of these quantities in the system and the rate of change of their quantity. We will sum up all flows further, considering the incoming flows as positive, and the outgoing flows as negative. We will divide the flows into convective and diffusive, marking the latter with the index *d*. Unlike the convective flow, the diffusion flow depends on the difference between the intensive variables of the system under study at the point where it enters or leaves and the intensive variables of the environment. In addition, we will use the following notations: *j*—flow index, $e_{j}$, $v_{j}$—internal energy and volume of one mole of the corresponding flow, and $P_{j}$—its pressure, $h_{j}=e_{j}+P_{j}v_{j}$—molar enthalpy, $h_{dj}$—enthalpy in the flow entering by diffusion, $q_{j}$—*j*-th heat flow, $N_{a}$—power produced by the system.

Let us present the general form of balance equations:

(3) $$\frac{dE}{dt}=\sum_{j}g_{j}h_{j}+\sum_{j}q_{dj}+\sum_{j}q_{j}-N_{a},$$

(4) $$\frac{dN_{i}}{dt}=\sum_{j}g_{j}x_{ij}+\sum_{j}g_{dj}x_{dj}+\sum_{\nu }\alpha_{i\nu }W_{\nu },$$

(5) $$\frac{dS}{dt}=\sum_{j}g_{j}s_{j}\sum_{j}\frac{q_{dj}-\sum_{i}g_{dj}\mu_{dij}}{T_{dj}}+\sum_{i\nu }\frac{\mu_{i\nu }n_{i\nu }}{T_{\nu }}+\sum_{j}\frac{q_{j}}{T_{j}}+\sigma .$$

Here $n_{i\nu }=-\alpha_{i\nu }W_{\nu }$ is the intensity of formation of the *i*-th substance in the ν-th reaction, $T_{\nu }$ is the temperature of the ν-th reaction. If there are no diffusion flows, then

(6) $$\frac{dE}{dt}=\sum_{j}g_{j}h_{j}+\sum_{j}q_{j}-N_{a},$$

(7) $$\frac{dN_{i}}{dt}=\sum_{j}g_{j}x_{ij}+\sum_{\nu }\alpha_{i\nu }W_{\nu },$$

(8) $$\frac{dS}{dt}=\sum_{j}g_{j}s_{j}+\sum_{j}\frac{q_{j}}{T_{j}}+\sum_{i\nu }\frac{\mu_{i\nu }n_{i\nu }}{T_{\nu }}+\sigma ,$$

where the number of heat flows includes heat flows released or absorbed during chemical reactions, which depend on the reaction rate.

If we consider a stationary mode of the process, i.e., when $dE/dt=dN_{i}/dt=dS/dt=0$, then the written equations from differential equations are transformed into final relations. When considering a cyclic process, balances can be written not for each moment in time, but on average for the cycle of the installation. Since the state of the system is the same at the beginning and end of the cycle, the total change in energy, amount of substance and entropy of the working fluid for the cycle are zero. In this case, balances are also reduced to a system of relations linking the average values of the terms on the right-hand sides of the equations for the cycle.

For **closed systems** consisting of several equilibrium subsystems, thermodynamic balances have the form

$${\dot{E}}_{0}=\sum_{i}{\dot{E}}_{i},\;{\dot{N}}_{0}=\sum_{i}{\dot{N}}_{i},\;{\dot{S}}_{0}=\sum_{i}{\dot{S}}_{i},$$

where *i* is the subsystem number, and index 0 refers to the system as a whole. In turn, ${\dot{E}}_{i}$, ${\dot{N}}_{i}$ and ${\dot{S}}_{i}$ are determined by the relations (3)–(5).

## 3. Processes of Minimal Dissipation

The requirement of minimum entropy production in processes of a given average intensity leads to the problem of such an organization of the process, in which the entropy production associated with it will be minimal [13]. Let us present a scheme for solving this problem for the scalar case.

Let two bodies contact, characterized by their extensive variables *Y* (volume, internal energy, entropy, number of moles of substance, …) and intensive *u* (temperature, composition, pressure, …). The difference in intensive variables leads to the appearance of an exchange flux *J*. The entropy increase over time *L* is equal to the integral of the product of the flux *J* and the driving force *X*, which, like *J*, depends on the intensive variables. Moreover, *J* and *X* always have the same sign. Dissipation is equal to the average rate of entropy increase. In this case, the intensive variables themselves depend on the extensive ones due to the equation of state. We will assume that the intensive variable $u_{2}$ of the second of the two contacting systems can be changed in an optimal way, and $u_{1}$ changes due to the influence of the exchange flow on it. The average value of the flow is given. The problem will take the form:

(9) $$\bar{\sigma }=\frac{1}{L}\int_{0}^{L}J\left(u_{1},u_{2}\right)X\left(u_{1},u_{2}\right)dl\to \min_{u_{2}\in V}$$

under conditions

(10) $$\frac{du_{1}}{dl}=\varphi \left(u_{1},u_{2}\right),\;u_{1}\left(0\right)=u_{10},$$

(11) $$\frac{1}{L}\int_{0}^{L}J\left(u_{1},u_{2}\right)dl=\bar{J}.$$

In these equations l is the duration of contact.

The value of *L* can be either fixed or subject to optimal selection.

The optimality conditions for this problem, as well as the optimality conditions for vector flows characterized by Onsager kinetics, are given in [14,15]. For the most important case, when

(12) $$\varphi \left(u_{1},u_{2}\right)=c\left(u_{1}\right)J\left(u_{1},u_{2}\right)$$

we obtain

(13) $$J^{2}\left(u_{1},u_{2}\right)=\lambda_{2}\left(\frac{\partial J(u_{1},u_{2})}{\partial u_{2}}:\frac{\partial X(u_{1},u_{2})}{\partial u_{2}}\right).$$

## 4. Form of the Realizability Region

**Figure 1:**
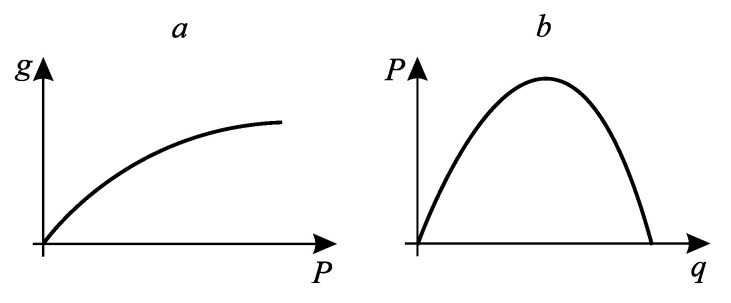
The form of the realizability set of a thermodynamic system for cases where the cost flow is mechanical or electrical energy (**a**) and when the cost flow is thermal energy (**b**). g is the target flow, P is the flow of expended (**a**) or produced (**b**) mechanical energy, q is the flow of heat.

Let the organization of processes in the system correspond to the conditions of minimal dissipation and the corresponding production of entropy $\sigma_{min}$, being substituted into the entropy balance equation, distinguishes the realizability region in the space of flows. The following statement is true [15]:

*If the system productivity monotonically depends on the flow of mechanical, electrical power, or separation power generated by the system, and the flow of costs monotonically depends on the heat flow, then the region of realized modes in the productivity-costs plane is limited from above (Figure 1a). If productivity is determined by the heat flow, and costs are determined by mechanical or electrical power, then productivity monotonically increases with increasing costs (Figure 1b)*.

The first type includes heat engines, rectification processes, absorption refrigerators, etc. The second type includes compression refrigerators and heat pumps, membrane separation systems, electric heaters, etc.

## 5. Maximum Work Problem

Let there be a non-uniform thermodynamic system consisting of several subsystems (reservoirs, subsystems of finite capacity), the intensive variables of which at the initial moment of time differ from each other. In addition, the system has a working body that can establish contact with each of the subsystems. The contact function U(t) takes the value of one in the presence and zero in the absence of contact. The coefficients of heat and mass transfer for such contact are limited. It is required to organize the contacts of the working body with the subsystems in such a way as to extract the maximum work from the system in a given time τ. The extracted work is equal to the change in the internal energy of the system, and since the initial state is given, the goal of the solution is the maximum decrease in the internal energy in a limited time. In this case, the state of the working body at the beginning and end of the process is usually assumed to be the same.

The result of solving the problem are two statements [16]:

*Statement 1: In a thermodynamic system consisting of reservoirs and a working fluid with a given initial state, for any laws of heat and mass transfer, the maximum work extracted during time τ corresponds to a process for which*

- *the vector of intensive variables u and contact functions U on the interval (0,τ) is piecewise constant, and the number of values it takes does not exceed r+m+2, where r is the number of conditions imposed on the final state of the subsystems and m is the dimension of the concentration vector;*
- *at the beginning and end of the process, the intensive variables of the working fluid change abruptly to some optimal values corresponding to optimal pressures;*
- *the entropy of the system grows on the interval (0,τ) as a piecewise linear function.*

Statement 1 follows from the properties of solutions of averaged problems presented in the previous section. Depending on the given boundary conditions, the maximum work can be greater or less than zero. In the latter case, it corresponds to the minimum of the expended work. We emphasize that such a structure of the optimal process is characteristic of any kinetics of heat and mass transfer.

*Corollary*: When no restrictions are imposed on the composition and entropy of the working fluid and the increments of the extensive variables of the reservoirs at t=τ, the entropy of the system in the optimal process for any laws of heat and mass transfer increases at a constant rate, and the working fluid contacts the same reservoirs throughout the process.

In the presence of subsystems of finite capacity, the problem of maximum work turns out to be an optimal control problem with integer variables U(t). In this case, it is true that

*Statement 2: On each interval of constancy of the contact function between the working fluid and the subsystem of finite capacity, the law of change of the vector u(t) of intensive variables of the working fluid in the optimal process must satisfy the conditions of minimal dissipation.*

## 6. Ideal Organization of Heat Exchange Systems

In multi-flow heat exchange systems with given input temperatures and heat capacities of hot flows, total heat exchange coefficient and heat load for Newton’s law of heat exchange, the minimum of entropy production corresponds to such an organization in which at each point of contact the ratio *m* of absolute temperatures of the heated and heating flows is the same, as is the temperature $\bar{T}$ of cold flows at the system outlet [17].

The following relationships are valid:

$$\left.\begin{array}{c} \bar{T}=\frac{\sum_{i}T_{i0}W_{i}-\bar{q}}{\sum_{i}W_{i}},\\ q^{*}\left(T_{i0}\right)=W_{i}\left(T_{i0}-\bar{T}\right),\\ \alpha^{*}\left(T_{i0}\right)=\frac{\bar{\alpha }W_{i}\left(\ln T_{i0}-\ln\bar{T}\right)}{\sum_{i}W_{i}\left(\ln T_{i0}-\ln\bar{T}\right)},\\ m=1-\frac{1}{\bar{\alpha }}\sum_{i}W_{i}\left(\ln T_{i0}-\ln\bar{T}\right),\\ {\bar{\sigma }}^{*}=\bar{\alpha }\frac{{\left(1-m\right)}^{2}}{m},\\ \alpha^{*}\left(T_{i0}\right)=q^{*}\left(T_{i0}\right)=W_{i}=0,\;T_{i0}\leq \bar{T}. \end{array}\right\}$$

Here $T_{i0}$, $W_{i0}$ are the temperature and heat capacity of the *i*-th hot stream, *q* is the heat flux, σ is the entropy production rate, and $\bar{\alpha }$ is the total heat transfer coefficient for the system. The last equality indicates that hot streams whose temperature does not exceed $\bar{T}$ should be excluded from the system.

## 7. Existence of the Irreversibility Index and Thermodynamic Analogy for Economic Macrosystems

In the economy, economic agents (EA) function at the micro level, each of which is uncontrollable. Together, they form a macrosystem that is largely analogous to the thermodynamic one [18].

### 7.1. Existence of the Welfare Function and Its Properties

The analogy between thermodynamic and microeconomic systems is obvious and has attracted the attention of many researchers [19,20,21,22]. The reason for this analogy is that both systems consist of a large number of individually uncontrolled elements, economic agents (EA), i.e., they are macrosystems. In order to make such an analogy strict, it is necessary to introduce a variable for microeconomic systems that is an indicator of the irreversibility of the processes occurring in them. The proof of the existence of such an indicator is given in [16]. Here it is given with minor abbreviations.

In exchange processes, the EA buys and sells resources, simultaneously changing the stocks of resources and capital. Let us introduce the function *U*, the differential of which

(14) $$dU=dM+\sum_{i}p_{i}dN_{i}.$$

We will call this function *capitalization* or *total capital* of the EA, since its change takes into account the change in both the basic resource *M* and the *tied capital* $F=\sum_{i}p_{i}N_{i}$. The vector *p* depends on the stocks of resources. The values $p_{i}$ have the dimension of the cost of the *i*-th resource in units of the basic resource.

Let some firm carry out an equilibrium exchange with the EA, exchanging some types of resources for others through purchases and sales. The exchange occurs reversibly and in such a way that the initial and final states of the EA in the space with coordinates $N_{i}$ coincide. If in such a process the firm could extract a certain amount of the basic resource, this would mean that the possibilities of its extraction are not limited, since it can be obtained only from one EA, without causing any changes in its state or in the state of the environment. From the impossibility of such an economic “perpetual motion machine” it follows that r=const

$$\oint \sum_{i}p_{i}\left(N,M\right)\;dN_{i}=0.$$

From this, in turn, it follows that there exists a function Q(N,r), whose partial derivatives with respect to $N_{i}$ are equal to $p_{i}$, and the differential has the form

$$dQ=\sum_{i}p_{i}\;dN_{i}+\frac{\partial Q}{\partial r}\;dr.$$

Here *r* is the valuation of the EA capital.

The expression (14) can be rewritten as

$$dU=dM+dQ-\frac{\partial Q}{\partial r}\;dr=d\left(M+Q\right)-\frac{\partial Q}{\partial r}\;dr.$$

Denoting M+Q=Y, and $-\frac{\partial Q}{\partial r}=\gamma$, we obtain that

(15) dU=dY+γdr.

Expression (15) depends on three variables: Y,γ and *r*. These variables are related to each other. If this were not so, then on the plane with coordinates Y,r it would be possible to move from a given initial state to any other with constant capitalization by choosing γ. In reality, the possibilities of such a transition are limited.

Indeed, let point 1 correspond to the initial state on the plane Y,r, and point 2 to the final state, such that with reversible exchange with the environment with a constant value of *r*, the capitalization of the EA decreases (dU<0). If it were possible to get to the same point at dU=0, then the EA could organize a cyclic exchange process in which its state would change from point 1 to point 2 along the trajectory with dU=0, and would return from point 2 to point 1 with a constant value of *r* and an increase in capitalization (dU>0). So it could increase its capitalization by any amount in a reversible exchange, which is impossible. Thus, γ is a function of *Y* and *r*. The differential of the total capital is thus a Pfaffian form of two variables, which always has an integrating factor.

Recall that a Pfaffian form is a first-order differential form, i.e., the sum of products of functions of several variables by the differentials of these variables,

$$dK=\sum_{i=1}^{n}F_{i}\left(x\right)\;dx_{i}.$$

If n=2 and the functions $F_{i}$ are differentiable, then there will always be an integrating factor f(x) such that dS=f(x)dK is a total differential, i.e., *S* depends on *x*, and ∮dS=0. The factor f(x) is usually not unique.

Before finding the integrating factor $p_{0}\left(Y,r\right)$, let us clarify the dependence of γ on these variables. To do this, consider a system consisting of two EAs with identical estimates of the basic resource $\left(r_{1}=r_{2}=r_{0}\right)$. Capitalization *U* and variable *Y* and the basic resource reserves for the system are additive, so that $U=U_{1}+U_{2}$, $Y=Y_{1}+Y_{2}$. So that $Q=Q_{1}+Q_{2}$. Accordingly, $\gamma =\frac{\partial Q}{\partial r}=\gamma_{1}+\gamma_{2}$. Thus, the dependence of γ on *Y* satisfies the superposition principle: $\gamma \left(Y_{1}+Y_{2},r\right)=\gamma \left(Y_{1},r\right)+\gamma \left(Y_{2},r\right)$ and has the form

(16) γ(Y,r)=G(r)Y+l(r),

Thus, for the capitalization differential we obtain the expression

dU=dY+(G(r)Y+l(r))dr.

The integrating factor can be any function $p_{0}$ such that

$$dS=p_{0}dU=p_{0}dY+p_{0}\left(G\left(r\right)Y+l\left(r\right)\right)dr$$

is a total differential. Since the mixed derivative of the function *S* does not depend on the order of differentiation, the function $p_{0}$ must satisfy the condition

(17) $$\frac{\partial S}{\partial r\partial Y}=\frac{\partial S}{\partial Y\partial r}\to \frac{\partial p_{0}}{\partial r}=\frac{\partial [p_{0}\left(G\left(r\right)Y+l\left(r\right)\right)]}{\partial Y}.$$

We will look for $p_{0}$ as a function of *r*, then the condition (17) leads to the equation

(18) $$\frac{\partial p_{0}}{\partial r}=p_{0}G\left(r\right).$$

One of the solutions to this equation is the function $p_{0}\left(r\right)=\exp\left[G\left(r\right)\right]$. It uniquely depends on *r* and can also serve as an estimate of the base resource.

Thus, there is some function of state (extensive variables) S(N,M) such that its differential has the form

(19) ∮dS=0.

In a process with non-zero flows, when prices do not coincide with estimates, the function *S* (the irreversibility index) always increases.

The estimates of resources can be expressed in terms of the function *S* as

(20) $$p_{0}=\frac{\partial S}{\partial M},\;p_{i}=\frac{\partial S}{\partial N_{i}}/\frac{\partial S}{\partial M},\;i=1,2,\ldots$$

In this case, the assessment of the basic resource $p_{0}$ is positive for all economic agents, and $p_{i}$ can be negative if the *i*-th resource requires, for example, utilization or storage costs. Cash can be considered one of the resources with a reserve of $N_{j}$, the assessment of which $p_{j}=1/p_{0}$. The function S(N,M) is called the welfare function or, more briefly, *welfare*.

When exchanging resources between economic agents, the conditions of voluntariness must be observed, which consist in the fact that none of the welfare functions $S_{\nu }$ decreases (the exception is associated exchange, charity). The conditions of voluntariness make it impossible to directly exchange one type of resource if its assessments by the EAs in contact with each other are of the same sign. Such an exchange becomes possible only if there is an intermediary.

If the welfare function is measured in national currency, then the value $p_{0}$ characterizes the value for the EA of the international currency and has the dimension [unit of national currency/unit of the basic resource]. The assessment $p_{0}$ of the basic resource in the processes of exchanging money by an economic agent on the foreign exchange market plays the same role as the assessment *p* in the exchange of resources.

The description of economic systems becomes formally close to the relations of thermodynamics if we introduce an *‘economic temperature’*,

$$T=\frac{1}{p_{0}}.$$

This notation was introduced in [23], the interpretation of this quantity, called *liquidity*, and its properties were given considerable attention in [24].

When the scale of the EA changes without changing its properties, the stocks of resources and capital are proportional to the scale. It is natural to assume that the welfare function changes in the same way, i.e., it is an extensive variable like *N* and *M*. In this case, the *S* function is homogeneous of the first order, and its derivatives with respect to *N* and *M* (estimates) are homogeneous functions of the zeroth order. According to Euler’s theorem for homogeneous functions, it can be written in the form

(21) $$S\left(N,M\right)=p_{0}\left(M,N\right)\left(\sum_{i}p_{i}\left(M,N\right)N_{i}+M\right).$$

The dependence p(N,M) can be found by formulas (20) and experimentally by the behavior of the EA in exchange processes.

If the existence of a welfare function *S* is postulated, then the estimates are determined through an extremal problem in which they enter as parameters:

(22) $$S\left(N,M\right)\to \max/\left(\sum_{i}p_{i}N_{i}+M\right)=U,$$

where the value of *U* is fixed.

The Lagrange function of the problem (22) is

$$L=S\left(N,M\right)-p_{0}\left(\sum_{i}p_{i}N_{i}+M\right),$$

where $p_{0}$ is an indefinite Lagrange multiplier. The stationarity conditions for *L* lead to the relations

(23) $$p_{i}\left(N,M\right)=\frac{\partial S}{\partial N_{i}}/\frac{\partial S}{\partial M},$$

which coincides with the expression (20). The function *S* is often assumed to be twice continuously differentiable, monotonically increasing in each of the arguments, strictly convex upward and equal to zero at the origin. Its partial derivatives tend to infinity when the corresponding variable tends to zero.

As a consequence of these assumptions, the solution to the problem (22) is unique and positive, and $p_{i}$ decreases with an increase in $N_{i}$. With this description, the EA is similar to a thermodynamic subsystem of finite capacity. For an economic reservoir, the resource estimates $p_{i}$ and $p_{0}$ are constant, and the welfare function is linear.

Note that in the general case, the welfare function of a system is not equal to the sum of the welfare functions of its constituent subsystems. Moreover, for each subsystem, the welfare function can have its own dimension. In contrast, the capitalization of each of the subsystems, the volume of the basic resource and the associated capital have the same dimension and their sum makes sense for the system as a whole under the conditions of internal equilibrium of each of the EA included in it.

### 7.2. Differential Relations Between Estimates, an Analogue of the Gibbs-Duhem Equation

Let’s write the differential of the function *S*,

(24) $$dS=p_{0}\left(dM+\sum_{i=1}^{n}p_{i}dN_{i}\right)=p_{0}dU.$$

Solving (24) with respect to dM, we obtain

(25) $$dM=\frac{dS}{p_{0}}-\sum_{i=1}^{n}p_{i}dN_{i}.$$

From equality (21) it follows that

(26) $$M=\frac{S}{p_{0}}-\sum_{i=1}^{n}p_{i}N_{i},$$

(27) $$dM=\frac{dS}{p_{0}}+Sd\left(\frac{1}{p_{0}}\right)-\sum_{i=1}^{n}\left(p_{i}dN_{i}+N_{i}dp_{i}\right).$$

Comparing equalities (27) and (25), we obtain a relationship linking the estimates of resources and capital

(28) $$Sd\left(\frac{1}{p_{0}}\right)-\sum_{i=1}^{n}N_{i}dp_{i}=0.$$

Similarly, comparing the differential *S* found from equality (21) with the expression (24), we obtain

(29) $$Mdp_{0}+\sum_{i=1}^{n}N_{i}d\left(p_{0}p_{i}\right)=0.$$

Conditions (28) and (29) follow from the existence of the function *S* and its homogeneity. They are economic analogues of the Gibbs-Duhem equations. For example, it follows from them that if the state of the system changes so that the resource estimates are constant, then the capital estimate $p_{0}$ is also unchanged.

Due to the symmetry of the matrix of second derivatives for a twice differentiable function, the sensitivity of estimates to changes in resource stocks and capital are related by the equalities

(30) $$\frac{\partial (p_{0}p_{i})}{\partial N_{j}}=\frac{\partial (p_{0}p_{j})}{\partial N_{i}}=\frac{\partial^{2}S}{\partial N_{i}\partial N_{j}},$$

(31) $$\frac{\partial p_{0}}{\partial N_{j}}=\frac{\partial (p_{0}p_{j})}{\partial M}=\frac{\partial^{2}S}{\partial M\partial N_{j}}.$$

From conditions (30) and (31) it follows that

(32) $$\frac{\partial p_{i}}{\partial N_{j}}+p_{i}\frac{\partial p_{j}}{\partial M}=\frac{\partial p_{j}}{\partial N_{i}}+p_{j}\frac{\partial p_{i}}{\partial M},\;i,j=1,\ldots ,n.$$

Equalities (30) and (31) are the economic analogue of Maxwell’s relations.

### 7.3. Dissipation of Capital

Let us return to the cyclical process of interaction of a firm with one EA and require that the average intensity of exchange processes be fixed. Then the firm when purchasing a resource will be forced to raise prices compared to the estimates $p_{i}$, and sell at prices that are lower than these estimates. The capitalization of the EA will increase, since

(33) $$\Delta U=\oint \sum \left(p_{i}\left(N,M\right)-c_{i}\right)\;dN_{i}\geq 0,$$

and the firm will suffer losses in comparison with the reversible process in the amount of ΔU.

We will call the intensity of the firm’s losses due to irreversibility,

(34) $$\sigma \left(t\right)=\sum_{i}n_{i}\left(p_{i},c_{i}\right)\left(p_{i}-c_{i}\right)\geq 0,$$

the *dissipation of capital* associated with the irreversibility of the resource exchange process. In this case, it has the meaning of trading costs.

The condition (33) of non-decreasing capitalization (and therefore welfare) in economic exchange is an analogue of the Clausius integral. Similarly, the law according to which at the contact of two EA, the resource passes from the EA for which its valuation is lower to the EA for which its valuation is higher, and at the same time the total value of the tied capital does not decrease $\left(\Delta \left(F_{1}+F_{2}\right)\geq 0\right)$, is an analogue of the second law of thermodynamics. This allows us to construct an irreversible microeconomics, in many ways similar to finite-time thermodynamics.

### 7.4. The Second Law of Microeconomics

The laws of conservation of matter and energy in microeconomics are the laws of conservation of resources. Here we will focus on the economic analogy of the second law of thermodynamics.

There are several formulations of the second law of thermodynamics, each of which can be considered a consequence of the others. Let us discuss the analogs of some of these formulations in microeconomics.

Among the numerous formulations of the second law, we will highlight two: the formulation of Clausius with Planck’s clarification: ≪*Heat by itself cannot pass from a cold body to a hotter body without leaving other changes*≫, and also the formulation of Leontovich: ≪*It is impossible to construct a device, as a result of the action of which positive work would be produced only due to the cooling of one body without any other changes*≫.

In microeconomics, the following statements correspond to the above formulations:

1. *A scalar resource flow cannot pass from an EA with a higher valuation to an EA with a lower valuation without leaving other changes.*
2. *It is impossible to extract capital by exchanging resources with one EA without any other changes.*

M. Planck formulated the following statement as a consequence of the second law of thermodynamics: ≪*In an irreversible process in a closed thermodynamic system, entropy can only increase and the exergy of the system can only decrease. The equilibrium state of such a system corresponds to the maximum entropy and the minimum performance under conditions corresponding to the imposed restrictions*≫. Similarly, for economic systems resource exchange processes in isolated microeconomic systems proceed in such a direction that the total tied capital of economic agents increases and reaches a maximum, and the potential ability of extracting the basic resource (profitability) decreases and reaches a minimum, under the restrictions imposed on the system, which include the conditions of voluntariness.

### 7.5. Analogies Between Thermodynamic and Microeconomic Systems and the Variables Characterizing Them

The notations adopted here and listed in Table 1 are: $T_{-}$ and *T*—temperatures of the reservoir and the system in contact with it, $p_{-}$—valuation of the resource on the market, *c*—price of the resource set by the monopolist firm, *N*—stock of the resource, *U*—internal energy of the system and total capital, *q* and *n*—flows of heat and resource, *M* and *F*—basic resource and tied capital.

### 7.6. Microeconomic Balances

#### 7.6.1. Open System

Let us write the balance equations for a heterogeneous economic system exchanging resource and capital flows with its environment. In this case, we assign index *i* to the *i*-th type of resource, and index *j* to the *j*-th subsystem. External resource and capital flows entering the system will be considered positive, and those leaving it will be considered negative. These flows are divided into two categories: those forced into the system and changing their intensity over time under the influence of external factors, and those dependent on prices set by external sellers and buyers, and on resource valuations in the subsystem under consideration. The first of these, by analogy with thermodynamics, will be called *convective* and marked with index *k*, and the second *diffusive* (index *d*). In addition, the subsystem can transform some types of resources into others.

The balance equation for the *i*-th resource is

(35) $$\dot{N_{i}}=\sum_{j}\left(n_{ij}^{k}\left(t\right)+n_{ij}^{d}\left(p_{j},c_{j}\right)+W_{j}\left(p_{j}\right)\alpha_{ij}\right),\;i=1,2,\ldots$$

Here the summation is carried out over all subsystems; $W_{j}\left(p_{j}\right)$—the intensity of the resource transformation process in the *j*-th subsystem, and the coefficients $\alpha_{ij}>0$ if the *i*-th resource arises in the *j*-th subsystem and $\alpha_{ij}<0$ if this resource is spent, they determine how much of the *i*-th resource arises (is spent) per unit of time; $c_{j}$—the price vector during the exchange of the *j*-th subsystem with the environment.

The balance equation for the basic resource is

(36) $$\dot{M}=\sum_{j}\left(m_{j}^{k}\left(t\right)-\sum_{i}c_{ij}n_{ij}^{d}\left(p_{j},c_{j}\right)\right)$$

and the balance sheet equation for tied capital is

(37) $$\dot{F}=\sum_{i,j}p_{ij}\left(N_{j},M_{j}\right)\left(n_{ij}^{k}\left(t\right)+n_{ij}^{d}\left(p_{j},c_{j}\right)\right)+\sigma ,$$

where capital dissipation σ is equal to

(38) $$\sigma =\frac{1}{2}\sum_{j}\sum_{\nu }n_{j\nu }\left(p_{j},p_{\nu }\right)\left(p_{j}-p_{\nu }\right)+\sum_{j}W_{j}\left(p_{j}\right)A_{j}.$$

Here $p_{j}$ and $p_{\nu }$ are vectors of resource estimates for the *j*-th and ν-th subsystems with components $p_{ij}$ and $p_{i\nu }$, $A_{j}=\sum_{i}\alpha_{ij}p_{ij}$, and $n_{j\nu }=-n_{\nu j}$ is a vector function of resource flow.

The first term in (38) is a change in associated capital due to resource exchange, and the second is due to the transformation of resources. $p_{j}$ and $p_{\nu }$ are vectors of resource estimates for contacting subsystems with components $p_{ji}$ and $p_{\nu i}$. Finally, $n_{j\nu }=-n_{\nu j}$—vector function of the exchange flow.

The value σ(p)≥0, so that in a heterogeneous open system in the absence of convective flows, the bound capital in the outgoing flows is not less than in the flows, entering the system. The equal sign corresponds to a homogeneous system.

As in thermodynamics, the condition $\sigma (p_{1},p_{2})\geq 0$ together with the balance relations (35)–(37) distinguishes the boundary of the feasibility region of an economic system in the class of reversible processes. The conditions imposed on the intensity of a particular flow allow us to determine the value $\sigma_{\min}>0$, achievable under these conditions. In this case, the area of feasibility narrows, since instead of the inequality σ≥0, the inequality $\sigma \geq \sigma_{\min}$.

If $\Delta p_{j\nu }=p_{j}-p_{\nu }$ is small, and the kinetic function $n_{i\nu }$ is differentiable with respect to the set of arguments, then σ is a positive-definite quadratic form of the variables $\Delta p_{i\nu }^{j}$. In the steady state, the right-hand sides of Equations (35)–(37) are equal to zero. In cyclic mode, when

N(0)=N(τ),M(0)=M(τ),F(0)=F(τ)

the integrals of the right-hand sides of these equations are equal to zero.

The condition of voluntary exchange imposes restrictions on the set of possible states of an open system. To find these restrictions, we write down the balance relations for the resource and capital for *j*-th subsystems;

(39) $$\dot{N_{ij}}=n_{ij}^{k}\left(t\right)+n_{ij}^{d}\left(p_{j},c_{j}\right)+\sum_{\nu }n_{ij\nu }\left(p_{j},c_{j\;nu}\right)+W_{j}\left(p_{j}\right)\alpha_{ij},i=1,2,\ldots$$

(40) $$\dot{M_{j}}=m_{j}^{k}\left(t\right)-\sum_{i}\left[c_{ij}n_{ij}^{d}\left(p_{j},c_{j}\right)+\sum_{\nu }c_{ij\;nu}n_{ij\nu }\left(p_{j},c_{j\nu }\right)\right].$$

Here $c_{j\nu }$ is a vector of intermediate prices with components $c_{j\nu i}$, which is determined from the continuity condition of flows,

(41) $${\tilde{n}}_{\nu ji}\left(p_{j},c_{j\nu }\right)=-{\tilde{n}}_{j\nu i}\left(c_{j\nu },p_{\nu }\right)=n_{\nu ji}\left(p_{j},p_{\nu }\right),\;i=1,\ldots ,m.$$

For any subsystem due to the conditions of voluntariness,

$$\dot{U_{j}}=\dot{S_{j}}/p_{0j}=\sum_{i}\left(p_{ij}\dot{N_{ij}}+\dot{M_{j}}\right)\geq 0,$$

which, taking into account (39) and (40), leads to restrictions on estimates and flows in the system:

(42) $$\begin{array}{c} \sum_{i}[p_{ij}\left[n_{ij}^{k}\left(t\right)+W_{j}\left(p_{j}\right)\alpha_{ij}\right]+\sum_{\nu }\left[n_{ij\nu }\left(p_{j},c_{j\nu }\right)\left(p_{ij}-c_{ij\nu }\right)\right]+\;+n_{ij}^{d}\left(p_{j},c_{j}\right)\left(p_{ij}-c_{ij}\right)]+m_{j}^{k}\left(t\right)\geq 0,\;j=1,2,\ldots .\; \end{array}$$

#### 7.6.2. Isolated System

For an isolated system, there are no external flows and the balance relations (35)–(37) take the form

(43) $$\dot{N_{i}}=\sum_{j}W_{j}\left(p_{j}\right)\alpha_{ij},\;N_{i}\left(0\right)=\sum_{j}N_{ij}\left(0\right),$$

(44) $$\dot{M}=\sum_{j}\dot{M_{j}}=0,$$

(45) $$\dot{F}=\sum_{j}\dot{F_{j}}=\sigma \geq 0.$$

In equilibrium, tied up capital is maximal, and the flows $n_{ij}$ and the rates of resource transformation $W_{j}\left(p_{j}\right)$ are equal to zero. In this case, the equilibrium distribution of the basic resource $\bar{M}$ depends on the kinetics of resource exchange between subsystems.

In thermodynamics, the equilibrium conditions can be obtained from the problem about the maximum of the total entropy of the system for a given total value of one or another extensive variable of the subsystems. For example, if the total volume of the subsystems is given, and the remaining extensive variables are unchanged, then the maximum of the total entropy corresponds to such a distribution of volumes, in which the derivatives of the entropy of each system with respect to its volume are the same, and this leads to equality of pressures. Similarly, the distribution of the total amount of thermal energy in equilibrium leads to equality of temperatures, etc.

In microeconomics, the conditions for the equilibrium distribution of resources can also be derived from the requirement of the maximum total tied capital of the system, given the total stock of resources. This leads to a distribution in which the derivatives of the tied capital of each subsystem with respect to the stock of its allocated resource (resource valuation) are the same.

For each *j*-th subsystems of the isolated system

(46) $$\dot{N_{ji}}=\sum_{\nu }n_{\nu ji}\left(p_{j},p_{\nu }\right)+W_{j}\left(p_{j}\right)\alpha_{ij},$$

(47) $$\dot{M_{j}}=-\sum_{\nu ,i}{\tilde{n}}_{\nu ji}\left(p_{j},c_{j\nu }\right)c_{j\nu i}\;\nu ,j=1,\ldots ,n.$$

Here $c_{j\nu }$ are intermediate prices determined by conditions (41). Thus, the price vector, and hence the right-hand side of the Equation (47) depends on the type of kinetics ${\tilde{n}}_{\nu j}$ of demand and supply of resources.

After determining from (41) the intermediate price $c_{j\nu }\left(p_{j},p_{\nu }\right)$ and substituting it into ${\tilde{n}}_{j\nu }$ and ${\tilde{n}}_{\nu j}$, these functions turn out to be the same and equal to the kinetic function with components $n_{j\nu }\left(p_{j},p_{\nu }\right)$, appearing in expressions (38) and (46).

As an example, let us consider the kinetics of the exchange of two EAs linear with respect to the difference between the price and the valuation and calculate the dissipation of capital σ in this case:

(48) $$n_{1}\left(p_{1},c\right)=a_{1}\left(p_{1}-c\right),$$

(49) $$n_{2}\left(p_{2},c\right)=a_{2}\left(p_{2}-c\right).$$

Let us find $c(p_{1},p_{2})$ using the condition $-n_{1}=n_{2}=n$:

$$a_{1}\left(p_{1}-c\right)+a_{2}\left(p_{2}-c\right)=0,$$

where

(50) $$c=\frac{a_{1}p_{1}+a_{2}p_{2}}{a_{1}+a_{2}},$$

(51) $$n\left(p_{1},p_{2}\right)=-n_{1}\left(p_{1},c\left(p_{1},p_{2}\right)\right)=\bar{a}\left(p_{2}-p_{1}\right),$$

(52) $$\bar{a}=\frac{a_{1}a_{2}}{a_{1}+a_{2}}.$$

Then the resource exchange dissipation is

(53) $$\sigma \left(p_{1},p_{2}\right)=\left(p_{2}-p_{1}\right)\bar{a}\left(p_{2}-p_{1}\right)=\bar{a}{\left(p_{2}-p_{1}\right)}^{2}=\frac{n^{2}\left(p_{1},p_{2}\right)}{\bar{a}}.$$

The equilibrium distribution of the basic resource $\bar{M}$ is determined by the exchange kinetics, and the equilibrium stocks of resources $\bar{N}$ also depend on $\bar{M}$, since they satisfy the equality

(54) $$p_{j}\left(\bar{M_{j}},\bar{N_{j}}\right)=p_{\nu }\left(\bar{M_{j}},\bar{N_{j}}\right)=\lambda ,\;\forall j,\nu .$$

In a number of cases, the set *Q* of values of the vector $\bar{M}$ that can be reached from a given initial state of the system for various functions $\tilde{n}\left(p,c\right)$ of demand and supply is of interest.

For each *j*-th subsystem, the minimum capital increase $\Delta M_{j}=\bar{M_{j}}-M_{j0}$ is achieved when the exchange with other subsystems is conducted at prices $c_{j\nu }$ arbitrarily close to $p_{j}$, i.e., reversibly, so that the value ${\bar{M_{j}}}^{\min}$ can be found by the condition $S_{j}\left(\bar{N_{j}},\bar{M_{j}}\right)=S_{j}\left(N_{j0},M_{j0}\right)$.

The maximum value of ${\bar{M_{j}}}^{\max}$ corresponds to an exchange at which $c_{j\nu }$ are arbitrarily close to $p_{\nu }$. Thus, in the space with coordinates $M_{j}$, it is possible to construct a parallelepiped with boundaries ${\bar{M_{j}}}^{\min}\leq \bar{M_{j}}\leq {\bar{M_{j}}}^{\max}$. The section of this parallelepiped by the plane

(55) $$\sum_{j}\bar{M_{j}}=\sum_{j}M_{j0}$$

selects the set *Q* of all possible equilibrium distributions of the basic resource between subsystems for different kinetic functions.

## 8. Generalization of Carathéodory’s Theorem and the Structure of Optimal Processes in Macrosystems

Exchange flows in macrosystems depend on intensive variables of subsystems, and the rate of change of extensive variables is determined by these flows. This leads to problems in which extensive variables are not included in the right-hand sides of differential equations that determine their change. For example, the change in the internal energy of two contacting subsystems is characterized by the differential equation

(56) $$\frac{dU_{1}}{dt}=q\left(T_{1},T_{2}\right).$$

The internal energy itself is not included in the right-hand side of this equation. If the values of the internal energy are subject to constraints $U_{1}\left(0\right)=u_{10},U_{1}\left(\tau \right)=u_{1\tau }$, then these constraints correspond to the condition imposed for a given value of τ on the average value of the current

$$\frac{1}{\tau }\int_{0}^{\tau }q\left(T_{1},T_{2}\right)=\frac{1}{\tau }\Delta U_{1}.$$

The Equation (56) are called Lyapunov-type equations by L.I. Rozonoer [3]. When solving problems, these equations can be discarded, replacing them with conditions imposed on the average rate of change of state variables, which leads to problems of averaged optimization. The same is typical for cyclically occurring processes in systems with intermediaries. In such systems, their efficiency indicators are averaged over a time equal to the duration of the cycle, which also leads to averaged problems. Below we present a proof of the theorem that defines the structure and type of optimal solutions to such problems.

### 8.1. On the Relationship Between Time Averaging and Set Averaging

The mean value of a continuous scalar function $f\left(x\left(t\right)\right),t\in \left[0,\tau \right],x\in V\subset R^{n}$ can be calculated over time as

(57) $$\bar{f_{t}\left(x\right)}=\frac{1}{\tau }\int_{0}^{\tau }f\left(x\left(t\right)\right)dt$$

or over a set as

(58) $$\bar{f_{p}\left(x\right)}=\int_{V}f\left(x\right)p\left(x\right)dx.$$

The function p(x) is called the distribution density. In the case where x(t) is a random function, p(x) is the distribution density of a random variable. It is non-negative and its integral on *V* is equal to one. In our case, x(t) is a deterministic function. Let us dwell in more detail on the properties of the function p(x), such that the results of averaging by Formulas (57) and (58) are the same.

We will assume that the variable *x* is scalar, the set *V* here and below is bounded and closed, and introduce the function $\theta \left(x_{0}\right),x_{0}\in V$, equal to the total duration of those time intervals *t* for which $x\left(t\right)\leq x_{0}$. Obviously, this function does not exceed τ. By $P(x_{0})$ we denote the ratio $\frac{\theta (x_{0})}{\tau }$, i.e., the fraction of the interval [0,τ] for which $x\left(t\right)\leq x_{0}$. This function increases monotonically with $x_{0}$, changing from zero to one. It is similar to the distribution function of a random variable.

The distribution density is

(59) $$p\left(x_{0}\right)=\frac{dP(x_{0})}{dx_{0}}=\frac{1}{\tau }\frac{d\theta (x_{0})}{dx_{0}}=\frac{1}{\tau }\frac{1}{\sum_{\nu }{\left|\frac{dx_{\nu }}{dt}\right|}_{x_{\nu }=x_{0}}}.$$

Here the interval θ increases with $x_{0}$ for any sign of the derivative for those values $x_{\nu }$ of the function x(t) for which it is equal to $x_{0}$.

If for some value $x_{0}$ the function x(t) is constant during a fraction γ of the interval [0,τ], then the function $P(x_{0})$ experiences a jump of magnitude γ at this point, and the distribution density at it is equal to $\gamma \delta (x-x_{0})$.

Example 1—Linear functions. Let $x\left(t\right)=\frac{ht}{\tau }$. Then by Formula (59) we obtain $p\left(x\right)=\frac{1}{h}=const$. The same distribution density corresponds to all triangles with base [0,τ] and height *h*.

Example 2—Piecewise constant functions. These functions take discrete values $x_{i}$, each during a fraction $\gamma_{i}$ of the interval [0,τ]. Any such function, according to Formula (59), corresponds to the distribution density

(60) $$p\left(x_{0}\right)=\sum_{i}\gamma_{i}\delta \left(x-x_{i}\right),\;\gamma_{i}>0,\;\sum_{i}\gamma_{i}=1.$$

The order in which a piecewise constant function assumes one or another of the possible values does not matter.

From these examples it is clear that to each function x(t) there corresponds a distribution density of its values p(x), defined on *V*, and to each distribution density there corresponds an arbitrary number of functions x(t), for which $\bar{f_{p}\left(x\right)}=\bar{f_{t}\left(x\right)}$. An exception is the distribution density of the form $p\left(x\right)=\delta \left(x-x_{1}\right)$. In this case, the corresponding function $x\left(t\right)=x_{1}=const$ on the entire interval [0,τ] and it is unique.

Let us consider the case when the function *f* depends on several, for simplicity on two, variables $x_{1}\left(t\right)$ and $x_{2}\left(t\right)$. In this case, the distribution function $P(x^{0})$ of the values of the vector *x* is a fraction of the interval [0,τ] for which two inequalities are satisfied: $x_{1}\left(t\right)\leq x_{1}^{0}$ and $x_{2}\left(t\right)\leq x_{2}^{0}$. This function monotonically increases with the growth of each of the arguments, when the first component of the vector $x^{0}$ is maximum ($p_{1}\left(x_{1}\right)=1$), it is equal to $P(x_{2}^{0})$, and its derivative is equal to the distribution density $p\left(x_{1}^{max},x_{2}\right)=p_{2}\left(x_{2}\right)$; similarly in the case when $x_{2}=x_{2}^{max},\;p\left(x_{2}^{max},x_{1}\right)=p_{1}\left(x_{1}\right)$. The functions $x_{1}\left(t\right)$ and $x_{2}\left(t\right)$ are independent of each other so that $p\left(x_{1},x_{2}\right)=p_{1}\left(x_{1}\right)p_{2}\left(x_{2}\right)$.

The desired solution to the averaged optimization problem is the distribution density $p^{*}\left(x\right)$ of the vector *x* on the set *V* of its admissible values. To implement this solution in time, one must find one of the possible functions x(t) that have the distribution $p^{*}\left(x\right)$. The solution to this last problem is significantly simplified by the features of the optimal solutions $p^{*}\left(x\right)$, proven in the next section.

### 8.2. On the Form of the Optimal Solution of Averaged Optimization Problems

We will denote the averaging operation by a bar drawn over the averaged function or vector. Thus,

$$\bar{x}=\int_{V}xp\left(x\right)dx,\;\bar{f(x)}=\int_{V}f\left(x\right)p\left(x\right)dx.$$

The simplest problem of averaged optimization is the problem of maximizing the average value of a scalar function f(x) for a given average value of its argument:

(61) $$\bar{f(x)}\to \max/\bar{x}=x_{0},x\in V\subset R^{n}$$

or in more detailed notation

(62) $$\int_{V}f\left(x\right)p\left(x\right)dx\to \max/\int_{V}xp\left(x\right)dx=x_{0},p\left(x\right)\geq 0,\int_{V}p\left(x\right)dx=1.$$

The unknown in this problem is p(x) (the distribution density of the vector of unknown variables). This function is non-negative and its integral on the set *V* is equal to one.

#### Carathéodory’s Theorem on Convex Hulls of Functions

Carathéodory’s theorem [25] on convex hulls of sets states that any element of the convex hull CoD of a compact set *D* in a Euclidean space of dimension *n* can be represented as an element of a simplex that has at most n+1 vertices (base points), each of which belongs to *D*.

In particular, a subgraph of a function f(x) can be a set *D*. The convex hull of a function is called the convex hull of a subgraph. A function that depends on *n* variables is the boundary of a set in the space $R^{n+1}$ of dimension *n*. The base points are certainly on this boundary, which means that their number does not exceed n+1. Below we will call Carathéodory’s theorem the theorem on convex hulls of functions.

The ordinate of the convex hull of the function $f_{0}\left(x\right)$ at the point $x_{0}$, which belongs to the convex hull of the set of definition of the function, is the value of the problem:

(63) $$\bar{f_{0}\left(x\right)}\to \max_{p(x)}/\begin{array}{c} \bar{x_{i}}=x_{i0},\;i=\bar{1,n},\\ x\in V\subset R^{n} \end{array}$$

where *V* is compact.

According to Carathéodory’s theorem, the optimal solution to this problem is

$$p^{*}\left(x\right)=\sum_{j=0}^{n}\gamma_{j}\delta \left(x-x^{j}\right),\;\gamma_{j}\geq 0,\;\sum_{i=0}^{n}\gamma_{j}=1.$$

That is, the optimal distribution is concentrated at no more than the (n+1)-th base point.

This fact allows us to rewrite the problem as a nonlinear programming (NP) problem:

(64) $$\sum_{j=0}^{n}\gamma_{j}f_{0}\left(x^{j}\right)\to \max/\begin{array}{c} \sum_{j=0}^{n}\gamma_{j}x^{j}=x_{0},\\ x^{j}\in V\subset R^{m},\;\sum_{j=0}^{n}\gamma_{j}=1,\;\gamma_{j}\geq 0. \end{array}$$

The variables are the base vectors $x^{j}$ and the vector of weight coefficients γ. We use the Kuhn-Tucker theorem [25] to solve it: If $y^{*}$ is a solution to a nonlinear programming problem

(65) $$f\left(y\right)\to \max/\varphi_{i}\left(y\right)\leq 0,\;y_{j}\geq 0,i=1,2,\ldots ,m,j=1,\ldots ,n,$$

then there exists a nonzero vector of factors

$$\lambda =\lambda_{0},\ldots ,\lambda_{m}\;\;\left(\lambda_{0}\;\text{is equal to}\;0\;\mathrm{or}\;1,\;\;\lambda_{i}\leq 0\;\mathrm{for}\;i>0\right),$$

such that for the Lagrange function

$$R=\lambda_{0}f\left(y\right)+\sum_{i=1}^{m}\lambda_{i}\varphi_{i}\left(y\right)$$

the following conditions hold:

(66) $${\left(\frac{\partial R}{\partial y_{j}}\right)}_{y=y^{*}}=0\;\mathrm{if}\;y_{j}^{*}>0;\;\;{\left(\frac{\partial R}{\partial y_{j}}\right)}_{y=y^{*}}\leq 0\;\mathrm{if}\;y_{j}^{*}=0,$$

and

(67) $$\lambda_{i}=0\;\mathrm{if}\;\varphi_{i}\left(y^{*}\right)<0;\;\;\;\lambda_{i}\leq 0\;\mathrm{if}\;\varphi_{i}\left(y^{*}\right)=0.$$

For problem (64), the Lagrange function has the form

(68) $$R=\sum_{j=0}^{n}\gamma_{j}\left[f_{0}\left(x^{j}\right)+\sum_{i=1}^{n}\lambda_{i}x_{i}^{j}-\Lambda \right],$$

where Λ is the Lagrange multiplier corresponding to the condition that the sum of the weight coefficients is equal to one.

The Kuhn–Tucker local unimprovability conditions with respect to weight coefficients lead to the requirements

(69) $$R^{0}\left(x_{j},\lambda \right)=f_{0}\left(x^{j}\right)+\sum_{i=1}^{n}\lambda_{i}x_{i}^{j}<\Lambda \;\;\mathrm{if}\;\gamma_{j}=0,$$

$$R^{0}\left(x_{j},\lambda \right)=f_{0}\left(x^{j}\right)+\sum_{i=1}^{n}\lambda_{i}x_{i}^{j}=\Lambda \;\;\mathrm{if}\;\gamma_{j}>0,\;j=0,\ldots ,n+1.$$

Here $R^{0}$ is the Lagrange function of problem (64) in the absence of averaging. Such a problem will be called *original*. Thus, we have

**Theorem** **1.** *For each of the basic values x included in the optimal solution to the problem of the convex hull of the function $f_{0}$ with a non-zero weight, the Lagrange function of the original problem is maximal, and the number of such points does not exceed n+1.*

### 8.3. Generalization of Carathéodory’s Theorem

In the NP problem with averaging of functions defining relationships between variables, it is required to achieve the maximum of the mean value of the function $f_{0}\left(x\right)$ on the set *V* of admissible values of *x*, provided that the mean value of the vector function $f\left(x\right)=(f_{1}\left(x\right),\ldots f_{i}\left(x\right),\ldots f_{m}\left(x\right))$ is zero. Formally

(70) $$\bar{f_{0}\left(x\right)}\to \max/\bar{f_{i}\left(x\right)}=0,\;i=1,\ldots ,m,\;x\in V\in R^{n},$$

yielding.

**Theorem** **2.** *1. The optimal distribution density in problem (70) has the form*

(71) $$p^{*}\left(x\right)=\sum_{j=0}^{m}\gamma_{j}\delta \left(x-x^{j}\right),\;\gamma_{j}\geq 0,\sum_{j=0}^{m}\gamma_{j}=1.$$

*2. There is a nonzero vector*

$$\lambda =\lambda_{0},\ldots \lambda_{i},..,\lambda_{m},\;\lambda_{0}=\left(0;1\right),$$

*such that at each base point $x^{j}$ the Lagrange function of the original problem*

(72) $$R=\sum_{i=0}^{m}\lambda_{i}f_{i}\left(x\right)$$

*is maximal with respect to x∈V.*

**Proof.**  To prove this statement, we introduce the concept of *reachability function* of the problem (70):

(73) $$f_{0}^{*}\left(C\right)=\max f_{0}\left(x\right)/f_{k}\left(x\right)=C_{k},\;k=1,\ldots ,m,\;x\in V.$$

This function is defined algorithmically on the set

$$V_{c}=\left\{C\in R^{m}:f\left(x\right)=C,x\in V\subset R^{n}\right\}.$$

It may not be smooth and upper semi-continuous.

The following is true: For those values of *x* for which f(x)=C, $p^{*}\left(x\right)$ is certainly equal to zero if $f_{0}\left(x\right)\neq f_{0}^{*}\left(C\right)$. Thus, the solution of the averaged problem can include with non-zero weight only those values of $x=x^{*}\left(C\right)$ for which the value of $f_{0}\left(x\right)$ coincides with the ordinate of the attainability function. Otherwise, it would be possible to change the distribution density so that the average value of $f_{0}\left(x\right)$ increases.

Since for each *C* the value $f_{0}$ coincides with the ordinate of the reachability function, problem (70) can be rewritten in the form

(74) $$\bar{f_{0}^{*}\left(C\right)}\to \max/\bar{C_{k}}=0,\;k=1,\ldots ,m,\;C\in V_{c}\subset R^{m}.$$

This is the problem of the ordinate of the convex hull of the reachability function at zero. According to the Carathéodory theorem on convex hulls, its optimal solution is

(75) $$p^{*}\left(C\right)=\sum_{j=0}^{m}\gamma_{j}\delta \left(C-C^{j}\right),\;\gamma_{j}\geq 0,\sum_{j=0}^{m}\gamma_{j}=1.$$

Since each basic value $C^{j}$ corresponds to the value $x^{j*}\left(C^{j}\right)$, the optimal distribution density in problem (70) has the form (71). Thus the first assertion of Theorem 2 is proved.

The proof of the second assertion completely repeats the analogous proof for the problem of the ordinate of the convex hull of a function, with the difference that the Lagrange function of the unaveraged problem has the form (72). We emphasize that the number of base points does not depend on the dimension of the vector *x*, but is determined by the dimension *m* of the vector function *f*. In the special case when $f_{j}\left(x\right)=x_{j}$, we obtain the theorem on the convex hull of a function.  □

Note that here and below, the conditions in the form of the maximum principle do not require the functions defining the averaged problem to be smooth in *x*; the set *V* may not be simply connected.

**Figure 2:**
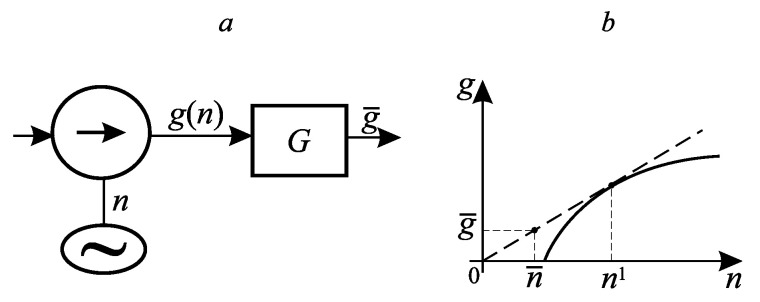
A system consisting of a pump and a smoothing tank (**a**); the dependence of the flow rate on the expended power (**b**).

**Example 1.** Consider a system consisting of an electric motor, a pump it rotates, and a tank. The motor consumes power *n* on which the pump performance *g* depends. The dependence g(n) is shown in Figure 2. It is required to find the mode for which, for a given average expended power $\bar{n}$, the average productivity $\bar{g}$ is maximum.

This is a problem about the ordinate of the convex hull of the function g(n) at the point $\bar{n}$. The number of base points is equal to two, one of them is the origin, and the second $n^{1}$, is determined by the condition of the maximum of the Lagrange function R=g(n)+λn at it, the same as at n=0. Eliminating λ from the conditions for the maximum of the Lagrange function and the requirement that this maximum be zero, we arrive at the equation for $n^{1}$:

$$\frac{g(n)}{n}=\frac{dg(n)}{dn}.$$

There are as many optimal implementations of this solution in time as you like. For each of them the pump power takes the value zero for one fraction of the period, and $n^{1}$ for the remaining fraction, and the fraction of the interval τ for which $n=n^{1}$ is equal to $1-\frac{\bar{n}}{n^{1}}.$ The maximum value of the interval τ is determined by the value of the capacity *G*, it is equal to

$$\tau_{max}=\frac{2G}{g(n^{1})}.$$

The value of the problem is

$${\bar{g}}^{*}=g\left(n^{1}\right)\left(1-\frac{\bar{n}}{n^{1}}\right).$$

It does not depend on *G*. When the capacity tends to zero, the sliding mode becomes the optimal solution.

### 8.4. Averaged Problem with Deterministic Variables

In averaged problems there can be two types of variables, random and deterministic. There is no averaging over the variables of the second type. Let us consider the NP problem, in which there is no averaging over some variables.

A problem with averaging over some variables will take the form

(76) $$\bar{f_{0}\left(x,y\right)}\to \max/\bar{f_{j}\left(x,y\right)}=0,\;\;x\in V\subset R^{n},\;\;y\in V_{y}\subset R^{K},\;\;j=1,\ldots ,m.$$

The functions $f_{0},\ldots ,\;f_{m}$ are continuous and continuously differentiable with respect to the set of arguments, the bar corresponds to averaging over x∈V, and the sets *V* and $V_{y}$ are closed and bounded.

For any *y*, this problem is an averaged nonlinear programming problem (70), and therefore, by Theorem 2, the optimal density function of *x* is concentrated at most at the (m+1)-th base point, so that $p^{*}\left(x\right)=\sum_{0}^{m}\gamma_{j}\delta \left(x-x^{j}\right)$, and there exists a nonzero vector λ such that at each of these points the Lagrange function of the original problem

(77) $$R=\sum_{j=0}^{m}\lambda_{j}f_{j}\left(x,y\right),\;\;x\in V\subset R^{n},\;\;y\in V_{y}\subset R^{K}$$

is maximal with respect to *x*.

The Lagrange function of problem (76), in which the density function *x* is $p^{*}\left(x\right)$, has the form

(78) $$\bar{R^{*}}=\sum_{j=0}^{m}\lambda_{j}\sum_{i=0}^{m}\gamma_{i}f_{j}\left(x^{i},y\right),\;\;x^{i}\in V\subset R^{n},\;\;y\in V_{y}\subset R^{K}.$$

For any density function p(x) of randomized variables, problem (76) is a nonlinear programming problem and, according to the Kuhn-Tucker theorem, there exists a nonzero vector λ with components $\lambda_{0}=\left(0;1\right),\;\lambda_{j},\;j=1,\ldots ,m,$ such that for the function (77) the optimal solution satisfies the conditions of local non-improvability with respect to *y*, in particular, the stationarity conditions

(79) $$\frac{\partial \bar{R^{*}}}{\partial y_{l}}\delta y_{l}\leq 0,\;\;l=1,\ldots K.$$

Here $\delta y_{l}$ is the admissible variation of $y_{l}$.

## 9. Conclusions

In FTT the specificity of problems on the optimal exchange of resources and problems with cyclically changing variables of the working fluid or mediator leads to averaged problems of nonlinear programming. Their optimal solutions are of a switching nature between the basic values of the variables. The maximum number of basic values is one more than the number of averaged conditions, and in each of them the Lagrange function of the non-averaged problem reaches a maximum.

The presented results of solving the problems of “Thermodynamics at finite time” illustrate the nature of its applications for systems of various natures, but do not pretend to be an exhaustive presentation. Not all the problems of FTT have been solved. In particular, the problem of the conditions of minimal dissipation for vector flows whose kinetics differs from Onsager’s awaits its solution. It is not clear what to consider the efficiency of a machine receiving energy from several sources. It is not clear what the irreversibility indicator looks like for macrosystems of a social nature, etc.

## Figures and Tables

**Table 1:** Equivalent nomenclatures for thermodynamic and microeconomic systems.

Thermodynamic System		Microeconomic System	
Name	Designation	Name	Designation
Reservoir (reversible heat exchange)	$T_{-}$	Economic reservoir	$p_{-}$
Reservoir (irreversible heat exchange)	$q=\alpha (T-T_{-})$	Monopoly market	$n=\alpha (c-p_{-})$
Amount of substance	*N*	Reserve of resource	*N*
System with finite capacity, chem. potential	μ(N)	EA, resource assessment	p(N)
Heat engine, temperature	T(t)	Intermediary firm, price	c(t)
Free energy, work	*A*	Basic resource	*M*
System performance	*E*	System profitability	*E*
System entropy	*S*	Tied capital	*F*
Entropy production	σ	Capital dissipation	σ
Internal energy	*U*	Total capital	U=M+F

## Data Availability

No new data were created or analyzed in this study. Data sharing is not applicable to this article.
